# Mobile Phone Apps for Inflammatory Bowel Disease Self-Management: A Systematic Assessment of Content and Tools

**DOI:** 10.2196/mhealth.4874

**Published:** 2016-02-01

**Authors:** Danny Con, Peter De Cruz

**Affiliations:** ^1^ Faculty of Medicine, Dentistry, and Health Sciences The University of Melbourne Melbourne Australia; ^2^ Department of Gastroenterology Austin Hospital Melbourne Australia; ^3^ Department of Medicine Austin Academic Centre The University of Melbourne Melbourne Australia

**Keywords:** IBD, apps, eHealth, smartphone, mhealth

## Abstract

**Background:**

The rising incidence of inflammatory bowel disease (IBD) over the past decade has resulted in increased health care utilization and longer IBD outpatient waiting lists. Self-management is recognized as an important aspect of chronic disease management but its application to IBD has been limited. The age of IBD onset in a majority of patients is in their 20s to 30s. Mobile phone apps are a technology familiar to young adults and represent an opportunity to explore self-management as a new model of health care delivery for IBD.

**Objective:**

The aim of this study was to explore the content and tools of existing IBD apps to identify functionalities that may facilitate patient self-management.

**Methods:**

We systematically assessed apps targeted at IBD patients via searches of Google (Android devices) and Apple (iOS devices) app stores with pre-defined inclusion and exclusion criteria. Apps were assessed for specific functionalities; presence of professional medical involvement; consistency with international IBD guidelines based on “complete,” “partial,” or “absent” coverage of consensus statements derived from the European Crohn’s and Colitis Organisation, American College of Gastroenterology, and the Gastroenterology Society of Australia; comprehensiveness of data that could be entered; and average pricing.

**Results:**

Of the 238 apps screened, 26 apps were assessed, including 10 available on Android platforms, 8 on iOS platforms, and 8 on both. Over half (14/26, 54%) of the apps had diary functionalities; over a third (10/26, 39%) provided health information about IBD. None of the apps offered decision support to facilitate the self-initiation of medical therapy. Five of 26 (19%) had professional medical involvement in their design. Apps demonstrated “complete” coverage of only 38% of the international consensus statements explored. The average price of the apps was AUD$1.37.

**Conclusions:**

Apps may provide a useful adjunct to the management of IBD patients. However, a majority of current apps suffer from a lack of professional medical involvement and limited coverage of international consensus guidelines. Future studies and app design for IBD should include professional medical involvement, evidence-based guidelines, and functionalities with decision support that are specifically tailored to patient self-management.

## Introduction

Inflammatory bowel disease (IBD) including Crohn’s disease (CD) and ulcerative colitis (UC) is a group of chronic inflammatory disorders of the intestine that have a relapsing and remitting disease course. IBD is associated with an increased prevalence of physical and psychological morbidity, and it adversely affects quality of life, societal interaction, and functioning [1,2]. Electronic health (eHealth) technologies incorporating self-management strategies to manage patients remotely may offer an effective alternative to classical outpatient-based approaches. However, the application of eHealth technologies to the IBD setting has been relatively limited [3-9]. The age of disease onset in the majority of patients is in their 20s and 30s [10,11]. Therefore the advent of mobile phones, a technology familiar to young adults, represents an opportunity to explore a new avenue of disease management in the form of mobile phone apps.

Apps, computer programs designed specifically to be run on mobile phones and tablet personal computers, are widely available to the consumer for download from online stores. The smartphone market is rapidly expanding. Nearly half (8.67 million) of Australian adults were estimated to be using a smartphone in May 2012, which increased to 64% (11.19 million) in May 2013 [12]. Over half (56%) of US adults owned a smartphone in 2013; specifically, 79% of adults aged 18-24 and 81% of adults aged 25-34 own smartphones [13].

The proliferation of mobile phones has facilitated the emergence of medical apps designed for management of chronic conditions such as asthma, diabetes, and rheumatoid arthritis [14-16]. These diseases are similar to IBD in that they are all characterized by chronicity requiring long-term pharmacological treatment and frequent outpatient clinic visits, with intermittent flares of disease activity requiring adjustments in medication. Apps for patients with these conditions typically have a number of common specified functions, including provision of disease information, dietary and lifestyle advice, electronic diaries for symptom tracking, and medication diaries and reminders. Some novel apps and mobile phone-based systems further provide functions for self-management of disease exacerbations [17-19]. Self-management whereby patients can adjust their therapy based on pre-determined algorithms or seek medical assessments is an emerging and promising aspect of chronic disease management that allows patients to maintain greater control over their disease [20-22].

Commercially available apps in the IBD setting perform many of the same functions as those that have been studied in asthma, diabetes, and rheumatoid arthritis, yet several apps in the IBD setting have not been subjected to adequate clinical evaluation and have been devised without taking into consideration current evidence-based guidelines. These apps have also been devised with limited professional medical involvement [23]. While the evidence supporting the utility of telemedicine and Internet-based interventions in IBD is emerging [5,7], the evidence behind the efficacy of mobile phone apps in the IBD setting has been lacking. Apps nonetheless represent the next logical step in our information technology era.

The purpose of this paper was to systematically study the content and functions of commercially available apps for IBD patients in the context of current clinical practice guidelines and discuss the results in relation to their utility in assisting patient with self-management of IBD.

## Methods

### Selection of Apps

We conducted a search of the official app stores of Apple (iOS) and Google (Android) for IBD-related apps on March 3, 2015. Search times included all of “IBD,” “crohn,” “crohns,” “colitis,” “ulcerative colitis,” and “inflammatory bowel disease.” Apps were screened by assessing their descriptions to exclude non-English apps and apps not targeting patients with IBD. We downloaded the remaining apps to test devices for further screening, based on predefined inclusion and exclusion criteria (see Table 1). Test devices included commercially available smartphones operating iOS and Android.

**Table 1:** Systematic search criteria.

Criteria	Description
Inclusion	Smartphone app
	Runs on iOS or Android operating systems
	Available for download from official app stores of Apple or Google
	English language
	Targets patients with IBD (as intended by the publisher)
	Free and paid apps
Exclusion	Requires invitation from publisher to use
	Targets patients with conditions other than IBD (as intended by the publisher)
	Does not target patients
	Unable to be tested due to technical difficulties

### App Assessment Criteria

Prior to the selection of apps, a preliminary search was conducted to identify the types of apps available for patients with IBD, which informed the development of assessment criteria that were used to assess the apps subsequently identified by this systematic review. Each app was independently assessed by 2 assessors. For all apps, we assessed for presence of professional medical involvement in the development of the apps, average ratings (out of 5), and number of reviews. Average ratings and number of reviews were available from both Google and Apple stores, so apps that were available on both platforms were documented twice. We also assessed the presence or absence of common functionalities for all apps, generated post hoc.

For diary apps, we assessed the comprehensiveness of four parameters that were able to be logged: abdominal pain, bowel habit, dietary intake, and medication. This was achieved by assessing the detail with which each symptom was able to be logged. For abdominal pain, these included the time and date, location, and severity. For bowel habits, these included time and date, consistency of stools, rectal urgency, presence of blood, and presence of mucus. For food diary apps, we assessed the extent to which time and date, type of food, quantity, and prior appetite were able to be logged. For medication, the time and date, medication name, and dosing were assessed. Where available, the input options for data entry were also assessed. For all parameters, we also included whether or not free-text notes were able to be entered.

For apps providing information related to IBD, we assessed the comprehensiveness and accuracy of the information provided. We formulated a set of statements extracted from and consistent with international guidelines for IBD from the European Crohn’s and Colitis Organisation [24-29], the American College of Gastroenterology [30,31], and the Gastroenterological Society of Australia [32]. A total of 14 statements was derived (see Table 2). Apps providing disease information were assessed to have either “complete,” “partial,” or “absent” coverage of each statement.

**Table 2:** Statements derived from international guidelines for IBD.

Topic	Criteria (Crohn’s disease)	Criteria (ulcerative colitis)
Overview	CD is a lifelong condition characterized by inflammation of the gastrointestinal tract	UC is a lifelong condition characterized by chronic inflammation of the colon
	CD has a relapsing and remitting course	UC has a relapsing and remitting course
	The causes of CD are unknown but are believed to be a mixture of genetic and environmental factors	The causes of UC are unknown but are believed to be a mixture of genetic and environmental factors
	The onset of CD is most common in the second and third decades of life	UC primarily presents in late adolescence and early adulthood
Diagnosis	CD is diagnosed by clinical evaluation and a combination of endoscopic, histological, radiological, and/or biochemical investigations	UC is diagnosed by clinical evaluation, proctosigmoidoscopy or endoscopy, biopsy, and negative stool examination for infective causes
	Chronic diarrhea is the most common presenting symptom of CD	Visible blood in the stools is the primary presenting symptom in UC
	Common symptoms of CD include chronic diarrhea, nocturnal diarrhea, abdominal pain, weight loss, fever, rectal bleeding	Common symptoms of UC include bloody diarrhea, rectal bleeding, and/or rectal urgency
Management goals	Goals of management in CD are the treatment of acute disease or induction of clinical remission, followed by maintenance of remission	Goals of treatment in UC are remission of symptoms, improved quality of life, reduction in long-term medication needs, and reduction of cancer risk
Treatment options	Initiation of therapy should be performed by a specialist gastroenterologist	
	GPs are important in monitoring long-term treatment plan	
	Therapy is divided into two categories: (1) acute therapy for flares to induce remission and (2) maintenance therapy in order to help maintain remission	
	Treatment for IBD may include pharmacological therapy and surgical therapy	
	Surgical therapy involves removal of a section of bowel, which may result in the patient living with a stoma for life	
	Use of complementary and alternative medicine is generally safe, but efficacy is not validated	

## Results

Searches of the official app store of Google (Android) yielded 146 apps available for screening. Of these, 32 apps were excluded as they were in a language other than English. Of the remaining 114 apps, 83 were excluded for either being unrelated to IBD or not being specific to IBD. A further 7 apps were excluded as they did not appear to target patients. The remaining 24 apps included 2 outdated versions of existing apps, 1 repeat app, and 3 apps that required invitation from the developers, which were excluded, leaving 18 Android apps for analysis.

Searches of the official app store of Apple (iOS) yielded 92 apps available for screening; 19 apps were excluded as they were in a language other than English and 46 of the remaining 73 apps were excluded for either not being related to IBD or not being specific to IBD. A further 9 apps were excluded for not targeting patients. Of the remaining 18 apps, 2 were excluded for requiring invitation from developers, leaving 16 iOS apps for analysis.

**Figure 1:**
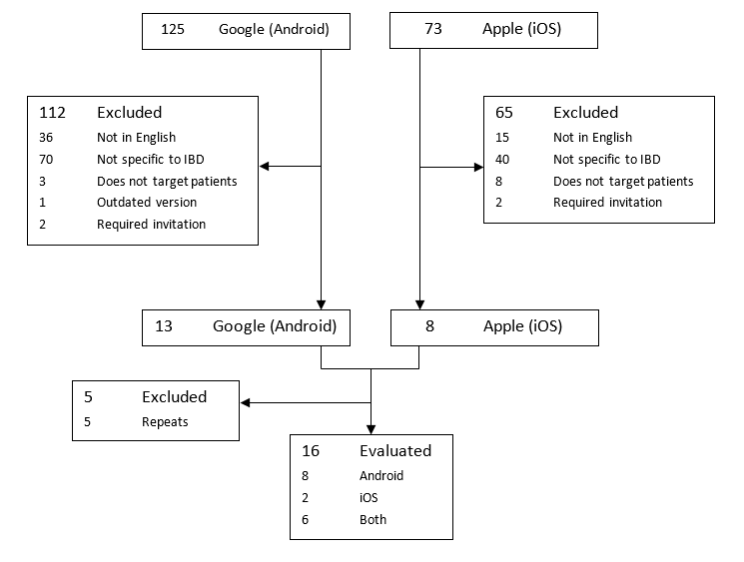
Flow diagram of search and selection process of apps.

The remaining 34 apps were downloaded and analyzed. Eight apps were subsequently excluded; 7 of which were duplicates, and one of which encountered technical difficulties in accessing the app, leaving 26 apps for analysis. A flow diagram of assessment of apps identified in this review is shown in Figure 1.

The final results included 26 mobile phone apps, of which 10 were available exclusively for Android platforms, 8 exclusively for iOS platforms, and 8 available for both. A summary of the results is shown in Tables 3 and 4 [33-58].

**Table 3:** Summary of assessed apps.

	Operating system	Professional medical involvement	Average rating (iTunes Store)	Ratings (iTunes Store), n	Average rating (Google Play)	Reviews (Google Play), n	Cost
AnswersIn Crohn’s Disease [33]	iOS	Yes	n/a	0	—	—	US $
AnswersIn Ulcerative Colitis [34]	iOS	Yes	4	1	—	—	US $
Colitis Diary [35]	Android, iOS		n/a	0	n/a	0	US $
Colitis Ulcerativa Manager [36]	Android, iOS	Yes	n/a	0	2	1	US $
Crohn’s Diary [37]	Android, iOS		n/a	0	n/a	0	US $
Crohn's Disease [38]	Android, iOS		n/a	0	n/a	0	US $
Crohn's Disease & Symptoms [39]	Android		—	—	n/a	0	Free
Crohn's Disease by AZoMedical [40]	iOS		n/a	0	—	—	Free
Crohn's Disease Manager [41]	Android, iOS	Yes	—	—	4.1	10	US $
Crohns Disease Symptoms & Suggested Treatment [42]	iOS		n/a	0	—	—	US $
CrohnsTracker Pro [43]	Android		—	—	n/a	0	US $
GI Buddy [44]	Android		—	—	3.2	63	Free
GI Monitor [45]	Android, iOS		3.3	42	3.9	954	Free
IBD [46]	iOS		2.9	15	—	—	Free
IBD (Crohn’s, Colitis) [47]	Android		—	—	3	21	Free
Lisa’s Diet [48]	iOS		n/a	0	—	—	Free
Living With Crohn's Disease [49]	Android		—	—	2.1	17	US $
My Crohn’s Diary (Android) [50]	Android		—	—	2.5	6	US $
My Crohn’s Diary (iOS) [51]	iOS		n/a	0	—	—	US $
MyCrohnsandColitisTeam Mobile [52]	Android, iOS		—	—	4.3	29	Free
myIBD [53]	Android, iOS	Yes	1	1	3	2	Free
Pentasa Timer [54]	Android		—	—	5	5	Free
Poocount [55]	Android		—	—	5	1	Free
Toilet diary [56]	Android		—	—	2.3	3	Free
Ulcerative Colitis Information [57]	Android		—	—	3.3	3	Free
Vualoo [58]	iOS		n/a	0	—	—	Free

**Table 4:** Functionalities of assessed apps.

	Bowel motion tracking	Pain tracking	Mood/psychological tracking	General symptom tracking	Dietary tracking	Medication tracking	Graphing/analysis	Community/social functions	Reminder system	Disease information
AnswersIn Crohn’s Disease [33]										Yes
AnswersIn Ulcerative Colitis [34]										Yes
Colitis Diary [35]	Yes	Yes	Yes	Yes	Yes	Yes	Yes			
Colitis Ulcerativa Manager [36]	Yes	Yes		Yes	Yes	Yes	Yes			
Crohn’s Diary [37]	Yes	Yes	Yes	Yes	Yes	Yes	Yes			
Crohn's Disease [38]										Yes
Crohn's Disease & Symptoms [39]										Yes
Crohn’s Disease by AZoMedical [40]										Yes
Crohn’s Disease Manager [41]	Yes	Yes		Yes	Yes	Yes	Yes			
Crohns Disease Symptoms & Suggested Treatment [42]										Yes
Crohns Tracker Pro [43]		Yes		Yes						
GI Buddy [44]	Yes	Yes		Yes	Yes	Yes		Yes		
GI Monitor [45]	Yes	Yes		Yes	Yes	Yes	Yes	Yes	Yes	
IBD (Crohn's, Colitis) [47]					Yes				Yes	Yes
IBD [46]										Yes
Lisa’s Diet [48]					Yes					
Living With Crohn’s Disease [49]										Yes
My Crohn’s Diary (Android) [50]				Yes	Yes	Yes				
My Crohn’s Diary (iOS) [51]			Yes	Yes	Yes					
MyCrohns andColitis Team Mobile [52]								Yes		
myIBD [53]	Yes	Yes		Yes	Yes	Yes	Yes			
Pentasa Timer [54]										
Poocount [55]	Yes						Yes			
Toilet diary [56]	Yes	Yes		Yes			Yes			
Ulcerative Colitis Information [57]										Yes
Vualoo [58]										

Five apps documented professional medical involvement in the development of the apps on the summary pages of the respective Google or Apple stores [33,34,36,41,53,59]. However, 2 of these apps stated only that “doctors” were involved, without specifying a particular person or organization [36,41]. One app was developed by a hospital [53]. Two apps appear to be original work by a gastroenterologist [33,34]. Most apps lacked app-store reviews, with only 7 apps having more than 10 reviews and only 1 app having more than 100 reviews. The latter app, however, had 954 reviews from the Google Play store [45]. Fourteen apps were free of charge, while the remaining 12 ranged from AUD$1.07-$8.22. When considering all 26 apps, the average cost was AUD$1.37 per app.

Thirteen apps exclusively had diary functionalities that allowed patients to track one of more aspects of their IBD. Of these, 9 apps presented only health information about IBD, 1 app had both, and 3 apps had neither. Of the 14 diary apps, 2 apps tracked only dietary intake, 3 apps tracked only disease symptoms, while the remaining 9 tracked both. Two apps had reminder functions, and both of these also had diary functions [45,47]. Eight apps were able to create graphs and/or tables to succinctly display logged information. Two apps were able to generate possible triggers for symptomatic episodes with a percentage likelihood based on previous user input [35,37]. One app [45] allowed photo sharing and communication on a message board. One app [52] was an adaptation of an online forum, where both app users and website users could post on the same message boards. One app [54] was utilized as a basic timer with no IBD specific functionalities but targeted patients with UC according to its description. One app allowed the tracking of calprotectin levels but was excluded from the analysis as it required an invitation from the developers.

### Apps Providing Disease Information

**Figure 2:**
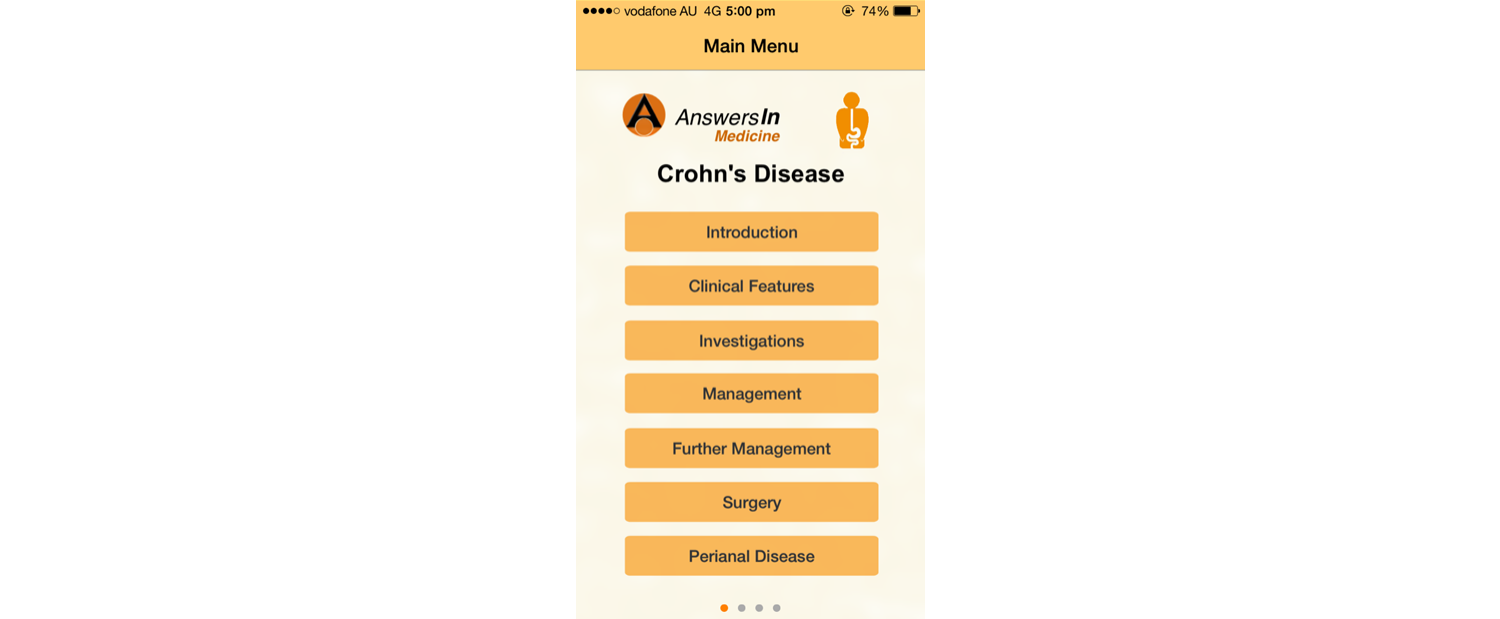
Screenshot of the main menu of the app AnswersIn Crohn’s Disease.

Ten apps provided information about Crohn’s disease, ulcerative colitis, or both. Nine of these apps provided information as their sole function, while 1 app also had diary functions [47]. However, the scope of the information provided in this app [47] was limited to the patient’s own experiences, while the diary function was limited to tracking only foods consumed without specifying time, quantity, or associated symptoms. Of the other 9 apps, 6 provided information on Crohn’s disease [33,38-40,42,49], 1 provided information on UC [57], and 2 provided information on IBD collectively [46,47]. Of the 6 Crohn’s disease apps, 1 displayed news and updates of latest research [40] and 1 app offered only dietary suggestions with no information about the disease itself [38]. Two apps, while comprehensive, were more suited for medical professionals [33,34]. An example of an app providing disease information is shown in Figure 2.

Only 2 apps providing disease information were created with professional medical involvement [33,34]. The information from 2 apps was discovered to have been derived from an online source, although the source location was not provided in either app [39,57]. One app was claimed to have been an original work and published as an e-book [49].

Eight information apps were evaluated for consistency with information from international IBD guidelines. Two of the 10 apps that included disease information were excluded from the analysis as one app included only news updates about IBD [40] while the other app included food suggestions for IBD without information about the disease itself [38]. We assessed the extent to which the IBD apps covered the 14 statements derived from international guidelines based on “complete,” “partial,” or “absent” coverage. Of the 112 potential instances where apps were assessed for one of the 14 identified statements, 42 (38%) statements demonstrated “complete coverage” and 30 (27%) statements demonstrating “partial coverage.” The remaining 40 (36%) statements demonstrated “absent coverage.”

### Diary Apps

#### Symptom Diary Apps

Summaries of the symptom diary apps for IBD found in this study are shown in Tables 5 and 6.

**Figure 3:**
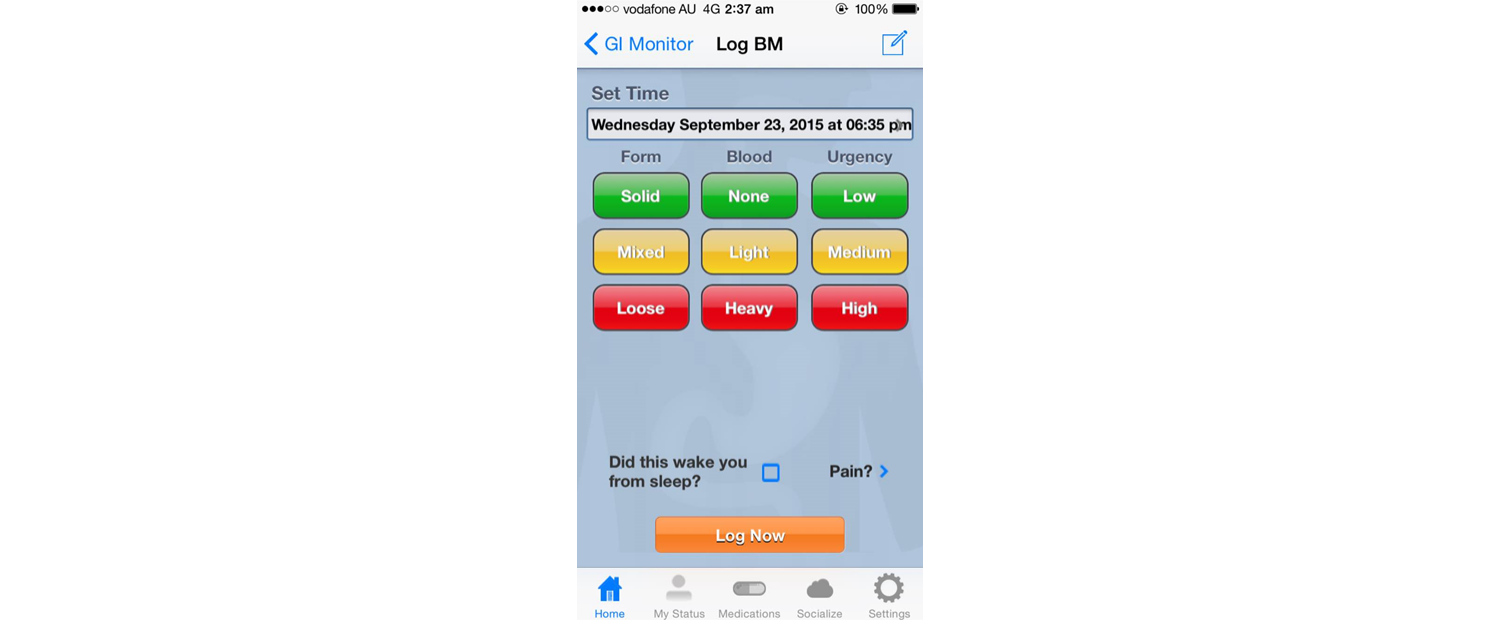
Screenshot of the interface of GI Monitor for recording bowel movements.

Although most of the symptom diary apps for IBD had the capacity to log multiple parameters, there was considerable heterogeneity regarding the detail with which symptoms were able to be recorded. Most of the symptom diary apps allowed the detailed recording of each bowel movement (including stool consistency and presence or absence of blood), but 2 apps [36,41] allowed only the logging of stool frequency. An example of a symptom diary app is shown in Figure 3. While most apps enabled recording of the specific times during which symptoms were experienced, 1 app instead elected to restrict the description of the timing of symptoms to a collective description of either daytime symptoms or nocturnal symptoms [53]. Four apps provided daily summary pages enabling a summary of symptoms over the course of a day, rather than the option of logging individual symptoms as separate new entries [35-37,41]. All apps except one [43] allowed users to input additional information in a “Notes” section.

**Table 5:** Apps offering bowel motion tracking.

	Time	Consistency	Urgency	Blood	Mucous	
Colitis Diary	Date and time	Multiple checkboxes (7 options)	Checkbox	Checkbox (“bloody stool”)	Checkbox (“mucus with stool”)	Blood and mucus are under “consistency”
Colitis Ulcerativa Manager	—	5-point scale (water, liquid, soft, normal, hard)	—	Checkbox (“blood within the stool”)	Checkbox (“slime within the stool”)	Can log defecation frequency per day
Crohn’s Diary	Date and time	Multiple checkboxes (7 options)	Checkbox	Checkbox (“bloody stool”)	Checkbox (“mucus with stool”)	Blood and mucus are under “consistency”
Crohn’s Disease Manager	—	5-point scale (water, liquid, soft, normal, hard)	—	Checkbox (“blood within the stool”)	Checkbox (“slime within the stool”)	Can log defecation frequency per day
GI Buddy	Date and time	3-point scale (formed, semi, liquid)	4-point scale (none, hurry, immediate, accident)	4-point scale (trace, blood, mucus, blood + mucus)	4-point scale (trace, blood, mucus, blood + mucus)	Blood and mucus are on same scale
GI Monitor	Date and time	3-point scale (solid, mixed, loose)	3-point scale (low, medium, high)	3-point scale (none, light, heavy)	—	
myIBD	Date and 2-point scale (day, night)	10-point sliding scale	10-point sliding scale	10-point sliding scale	—	
Poocount	Date and time	—	—	3-point scale (blood 0, blood +, blood ++)	—	
Toilet diary	—	Checkbox (“diarrhea”)	Checkbox (“enormous pressure”)	Checkbox (“blood”)	Checkbox (“mucus”)	

**Table 6:** Apps offering pain tracking.

	Time	Severity	Location	
Colitis Diary	Date and time	10-point drop down menu	—	Includes a dropdown menu for “pain description”
Colitis Ulcerativa Manager	Date and time	10-point sliding scale	—	
Crohns Diary	Date and time	10-point drop down menu	—	Includes a dropdown menu for “pain description”
CrohnsTracker Pro	Date	—	—	
Crohn’s Disease Manager	Date and time	10-point sliding scale	—	
GI Buddy	Date and time	4-point scale (none, mild, moderate, severe)	—	
GI Monitor	Date and time	10-point sliding scale	—	
myIBD	Date	10-point sliding scale	Picture-based	User is able to tap on arbitrary location on picture of abdomen
Toilet diary	—	—	—	Only has checkbox for presence of “cramp”

#### Food Diary Apps

A summary of the food diary apps found is shown in Table 7.

**Figure 4:**
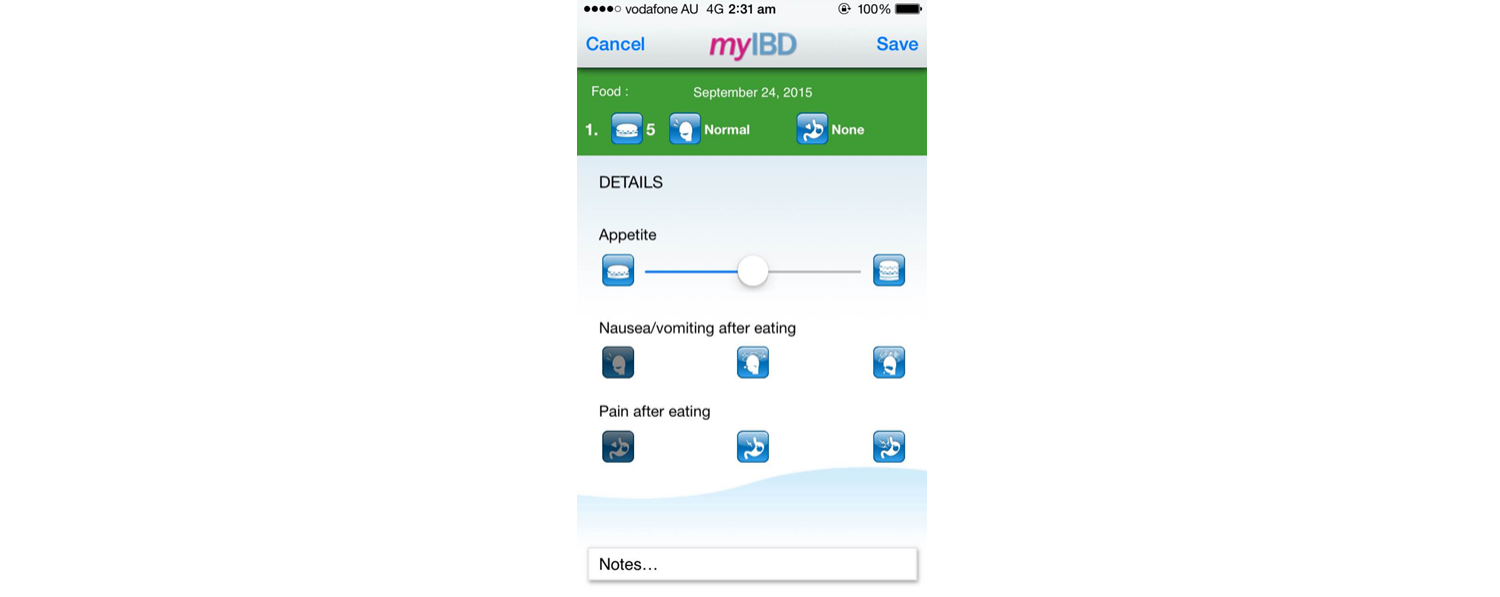
Screenshot of the interface of myIBD for the recording of dietary intake.

Most diary apps were able to record both symptoms and dietary intake, except 2 apps [47,48], which were able to record only dietary intake. An example of a food diary app is shown in Figure 4. Inconsistencies were present in the ability of apps to log the date and time of meals. One app [47] offered only the ability to list “good foods” and “foods to be avoided” without the capacity to log food intake throughout the day. Five apps enabled logging of daily food intake but did not include the timing of food intake. Most apps offered only free-text input for the names of food; however, 2 apps [44,48] had a search function to select predefined foods. Although some apps in this study provided a search function or dropdown menu [35,37,43,44,50], the list of food items was often not comprehensive. One app used the smartphone camera to take pictures of meals as they were logged [48]. Two apps only logged meals in the form of a free-text input section titled “influence of food” as part of a broader diary entry [36,41]. Two apps recorded the serving size of each food item [44,50]. One app recorded the appetite of the user prior to consuming the food [53]. Eight apps allowed users to type free-text notes to record additional information regarding each meal.

**Table 7:** Apps offering dietary tracking.

	Time	Food item	Serving size	Appetite	Note function	Additional features
Colitis Diary	—	List menu OR free input	—	—	Yes	Can log other triggers such as “skipped a meal”
Colitis Ulcerativa Manager	—	—	—	—	Yes	
Crohns Diary	—	List menu OR free input	—	—	Yes	Can log other triggers such as “skipped a meal”
Crohn’s Disease Manager	—	—	—	—	Yes	
CrohnsTracker Pro	Date	List menu OR free input OR saved items	—	—	—	
GI Monitor	Date and time	Free input	—	—	—	Can log perceived difficulty of digesting food
GI Buddy	Date and option of breakfast, lunch, dinner, snack	Search function OR free input OR saved items	Yes	—	—	Can set custom meals
IBD (Crohn’s, Colitis)	—	Free input	—	—	—	Allows logging of “good foods” and “foods to avoid”
Lisa’s Diet	Date and time	Drop down menu OR free input OR saved items	—	—	Yes	Can search for foods flagged by other users
My Crohn’s Diary (Android)	Date and time	Drop down menu OR free input	Yes	—	Yes	Can log fluid intake separately
My Crohn’s Diary (iOS)	Date	Free input	—	—	Yes	Can log fluid intake separately
myIBD	Date	—	—	Yes	Yes	Can log associated nausea and pain levels

### Management Apps

#### Decision Support Apps

At present, no apps offering decision support specific to self-initiation of therapy were identified by this study. However, the app, GI Monitor, was able to automatically generate a quality-of-life score based on patient-reported outcomes collected via the diary function [45].

#### Reminder Apps

While none of the apps found used a reminder system as their primary functionality, 2 apps had medication reminder functions [45,47]. Both of these apps also had diary functions. However, one of these lacked any useful ability to track symptoms or dietary intake [47].

## Discussion

### Principal Findings

Mobile phones represent a promising tool to facilitate self-management in chronic disease. Self-management aims to give a degree of disease control back to the patient by improving self-efficacy, knowledge, and understanding of their disease. Despite their appeal, the evidence regarding the role of apps in self-management of IBD is limited and their optimal use to facilitate self-management is yet to be determined.

This systematic review utilized a systematic approach to explore the content and tools of commercially available mobile phone apps for patients with IBD. However, a lack of standardized assessment criteria for eHealth modalities such as apps precluded a specific rating and/or ranking of the utility of the apps by the reviewers. Although the user ratings of the apps were included in this review, they are a somewhat subjective representation of the apps’ quality of content and functionalities. Ratings and total number of ratings are likely to demonstrate the popularity or perceived value by patients, which can be influenced by advertising or referral (such as a health service “prescribing” a particular app). We acknowledge that a mobile app rating scale has been proposed as a potential method of assessing apps, but such a scale is yet to be validated [60]. This review highlights the need for consensus regarding regulation and evaluation of eHealth technologies if mobile phone apps and other eHealth modalities are to be integrated into mainstream medical practice.

In our assessment, apps providing disease information demonstrated extensive coverage of various topics in IBD, including disease overview, causes, symptoms, diagnosis, and treatment. Although some of these topics were explored in depth, the apps failed overall to provide complete coverage of many of the evidence-based statements derived from international guidelines. This indicates that the majority of apps were not sufficiently accurate or comprehensive as patient education tools, which is most likely explained by the lack of professional medical involvement in their development. In this study, although we anticipated that a proportion of the apps would lack professional medical involvement in their design, we did not expect that this would exceed three quarters of the apps identified. Medical professionals should be consulted in the development process of apps to help ensure that relevant, evidence-based information is included within patient education tools. A lack of evidence undermines the safety and quality of mobile phone apps and thus may pose risks to patients [61]. Peer-reviewing medical apps represents another potential solution to quality assurance, but its feasibility is unknown [62]. Ultimately it is important to ensure that patient safety is not compromised, especially if the app is to be recommended or “prescribed” by a doctor.

Despite the theoretical importance of professional medical involvement, adherence to evidence and app regulation, mobile phone apps that are not used are ultimately ineffective. The challenge is to balance the safety and quality requirements of medical mobile phone apps with the design features required to promote adherence to therapy. Uptake and usage of mobile and Internet technology are influenced by an array of factors, such as perceived risk of use, perceived usefulness, and degree of user-centric focus, which have been described in behavioral change models in emerging eHealth literature [63-65]. Addressing adherence to management is a necessary prerequisite alongside the quality and safety requirements of apps as potential medical devices. Therefore, future app developments should have strong grounding in behavioral change theory and place considerations in predictions of usage and user-centric approaches. A development framework devised for Internet interventions may be applied to the mobile phone app setting to integrate the magnitude of complex design features [66].

App content is also important in improving adherence and plays the most significant role in effecting behavioral change. This review identifies a number of tools and functionalities in existing apps that may be beneficial. Patient education in IBD is important to enable everyday behaviors that promote well-being and prevent deterioration [67]. Volitional non-adherence, which is prevalent in IBD and contributes to greater disease activity, reduced quality of life, and greater health care costs [68-75], may be addressed by adequate provision of disease information. Patients who are better informed about their condition and medications are more likely to adhere to their prescribed treatment regimens [76,77]. An ideal patient education app provides appropriate evidence-based information that effectively meets the educational needs of patients.

Prior to the advent of the smartphone, SMS (short message service) text messaging provided a method for medication reminders using traditional mobile phones. In controlled environments, reminder text messages have been shown to decrease the incidence of missed medication doses and improve adherence to treatment [74,78] and there is some research to suggest that reminders have a dose-response effect [79]. Apps that provide a reminder functionality may be useful in enhancing compliance in poorly adherent patients [80,81]. Compared with SMS, apps may offer additional interactivity, customizability, and functionality. However, the usefulness of smartphone reminder apps in practice remains to be seen and studies exploring reminder text messages have been limited to controlled environments with small sample sizes. Future studies should explore the efficacy and dose-response relationship of reminder apps in large populations consisting of patients who are non-adherent due to involuntary factors such as forgetfulness.

Mobile phone apps with diary functionalities are emerging as a potential successor to traditional paper-based diaries due to their improved accessibility and convenience. Despite their feasibility being demonstrated in several other chronic disease settings [14,16,23,82,83], there have been no completed studies that have investigated commercially available IBD diary apps. However, the self-management app, “Health PROMISE”, as of May 2015 is being investigated for its effects on various outcome measures including quality of life, number of emergency visits, and number of hospitalizations in an IBD cohort as part of a multicenter study [21]. Furthermore, while not a smartphone app, a recent study found that Web-based diaries (using the Harvey-Bradshaw index) were effective in the monitoring of clinical disease activity in patients with Crohn’s disease with good correlation demonstrated with the more widely accepted Crohn’s disease activity index [84]. The benefit of symptom diaries is that they allow patients to maintain a degree of control over their condition in the setting of alterations in disease activity and medications, and in doing so, may help increase engagement of patients in their management and facilitate adherence. However, while previous studies have shown electronic diaries to be feasible in controlled environments, there has been little evidence supporting the usage and efficacy of commercially available electronic diaries. Studies to evaluate the optimal use of diary apps in the assessment and monitoring of IBD patients are therefore required.

Symptom diaries may have further utility when combined with food diaries as a means of identifying potential dietary triggers for gastrointestinal symptoms in IBD [85-88]. Self-initiated food avoidance is highly prevalent in patients with IBD [89]. The use of food diaries has consequently been recommended by the Crohn’s & Colitis Foundation of America [90,91]. Symptom diaries can therefore be correlated with particular meals to help identify food precipitants and can be reviewed by dieticians to help guide food selection and avoid unnecessary caloric restriction. Moreover food diaries may help identify patients at risk of malnutrition and nutritional deficiencies, which are more prevalent in IBD patients [89,92].

Programs that provide decision support to the patient are emerging as promising management options in IBD [20,22]. Decision support apps use patient-reported outcomes collected via diary functionalities to generate individualized management plans for the patient. However, none of the apps studied in this review contained specific information regarding alarm symptoms and none of the apps provided specific feedback to patients in the event of an escalation of symptoms, thereby potentially limiting their utility in self-management. Although the efficacy and feasibility of apps in facilitating self-initiation of therapy is yet to be proven [93], apps that provide decision support for patients are currently in development and are likely to be regulated in the United States and United Kingdom as medical devices, rather than commercial programs [21,94,95]. The implications are unclear, but regulatory guidelines for the development and evaluation of such apps are required.

Although apps that have been specifically developed for self-management of IBD are lacking, evidence from other chronic diseases suggests that they may have a role in improving self-efficacy, knowledge, and understanding and enhance adherence to prescribed therapy. Developing a comprehensive self-management app may mark the important first steps in propagating self-management in IBD in a similar vein to the creation of asthma management plans for asthma self-management. Apps may potentiate the self-initiation of therapy in acute IBD flares by first measuring disease activity using a symptom diary and providing appropriate subsequent management advice. A novel asthma self-management program with natural language recognition capabilities was recently found to promote medication adherence and patient confidence over the course of its trial [18]. The program allowed adolescents to communicate with the system via text messaging, emulating conversation with a clinician. It was able to interpret patient descriptions of asthma exacerbations and generate management plans using a predefined algorithm. A diabetes self-management app similarly incorporated an algorithm to calculate the required insulin bolus amount depending on blood glucose levels before meals, carbohydrate counts, and planned physical activity that were manually entered into the program [17]. These apps not only provide treatment advice in critical times where professional consultation is not readily available but also increase the control that patients have over their own illnesses. While these eHealth solutions have demonstrated some efficacy in controlled environments, larger population-based studies are required to supply more reliable evidence for their use in clinical practice.

Although a Web-based approach to IBD management has been demonstrated to improve patient engagement, quality of life, and reduce the duration of relapse, there are currently no data supporting the efficacy of IBD apps in self-management [22]. Gastroenterologists have also suggested that digital tools should focus on promoting patient compliance with treatment, with reduced emphasis on patient-physician concordance [96]. Reaching a compromise between empowering patients with shared decision making and encouraging patients to follow instructions to improve compliance will require discussions between patients and their clinicians. However, it remains to be established as to whether self-management apps that improve patient autonomy will have a positive or negative impact on treatment compliance.

### Limitations

The main limitation of this systematic review was that the assessment method for the apps was developed by the authors and has not been validated. This was necessary due to a lack of published studies on IBD apps, as well as a lack of end-user data to allow a quantitative assessment of the efficacy of the apps. Furthermore, the formal assessment of the identified apps did not take design or usability into account. This systematic review was also limited to English-language apps for the two most popular operating systems, Android and iOS. Despite the limitations of this review, we believe that it has provided a real-world exploration of the IBD apps that are currently available and their strengths and limitations respectively.

### Conclusion

Apps may provide a useful adjunct to the monitoring and management of patients with IBD. Current apps available for IBD offer varying degrees of content, functions, and levels of care but do not offer patients decision support for their IBD self-management. IBD apps currently suffer from a lack of professional medical involvement, adherence to international clinical guidelines, and validation from clinical studies. These limitations jeopardize the safety and quality of apps as potential medical tools from the clinician’s perspective. The design process is complex and needs to reconcile the needs of clinicians with design features that are desired by patients. Despite their current limitations, apps have the potential to become useful tools to implement clinical pathways and algorithms to support decision making for patients and engage patients in taking an active role in their care. Future mobile phone apps should use behavioral change models in their development to improve uptake and adherence. Future studies should explore the perceived needs of patients and clinicians in relation to apps in the IBD setting. Such information would inform the development of quality apps that meet the needs of both patients with IBD and their treating clinicians. Further clinical testing is required before the routine recommendation of mobile phone apps to support the management of IBD becomes feasible. Ultimately, it is unlikely that a single app will fulfill the needs of all patients with IBD, but more likely that clinicians will prescribe a range of apps each with a specific purpose tailored to the relevant needs of each individual IBD patient.

## Abbreviations

CD: Crohn’s disease
GESA: Gastroenterological Society of Australia
IBD: inflammatory bowel disease
SMS: short message service
UC: ulcerative colitis
